# Individuals with Alzheimer’s disease living alone in Sweden receive limited comprehensive care

**DOI:** 10.1002/alz.087599

**Published:** 2025-01-09

**Authors:** Dorota Religa

**Affiliations:** ^1^ Division of Clinical Geriatrics, Center for Alzheimer Research, Department of Neurobiology, Care Sciences and Society, Karolinska Institutet, Stockholm, Sweden; Theme Inflammation and Aging, Karolinska University Hospital, Stockholm Sweden

## Abstract

**Background:**

A significant number of individuals with Alzheimer’s disease (AD) reside independently in their homes, yet there is limited understanding of whether they receive comparable diagnostic and treatment standards for AD compared to their cohabiting counterparts. The prevalence of dementia is rising, particularly among the elderly, with approximately 150,000 individuals affected by dementia in Sweden, two‐thirds of whom have AD. The national quality registry SveDem (Swedish registry for cognitive (dementia disorders) was established to promote consistent and high‐quality dementia care across the country.

**Methods:**

Conducted as a cross‐sectional cohort study utilizing data from the Swedish registry for cognitive/dementia disorders (SveDem), we focused on patients in the community diagnosed with AD between 2007 and 2015 (n = 26,163). Information on medications and comorbidities was extracted from the Swedish Prescribed Drug Register and the Swedish Patient Register.

**Result:**

Among the study participants, 46% (11,878) lived alone, primarily comprising older women. After adjusting for confounding factors, living alone was associated inversely with the likelihood of receiving computed tomography (OR 0.90; 95% CI 0.82‐0.99), magnetic resonance imaging (OR 0.91; 95% CI 0.83‐0.99), and lumbar puncture (OR 0.86; 95% CI 0.80‐0.92). Additionally, living alone was negatively correlated with the utilization of cholinesterase inhibitors (OR 0.81; 95% CI 0.76‐0.87), memantine (OR 0.77; 95% CI 0.72‐0.83), and cardiovascular medication (OR 0.92; 95% CI 0.86‐0.99). Conversely, living alone was positively associated with the use of antidepressants (OR 1.15; 95% CI 1.08‐1.22), antipsychotics (OR 1.41; 95% CI 1.25‐1.58), and hypnotics and sedatives (OR 1.09; 95% CI 1.02‐1.17). When mew data on other dementia were included: among the study participants up to 2018 (n = 80,004), 43.8% (35053) lived alone, primarily comprising older women (58.6%). The mean age was 79.6 (SD 7.9). The following age groups were included under 70 8456 (10.6%), 70‐79 27381 (34.2%), 80‐89 37628 (47.0%) and 90+ 6539 (8.2%).

**Conclusion:**

Individuals with AD living alone receive less comprehensive care compared to their cohabiting counterparts. Finding will be presented on other types of dementia.

